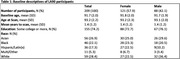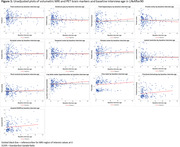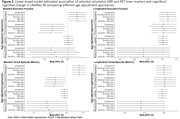# Age adjustment approaches to estimating the association of neuroimaging biomarkers and cognition in the oldest‐old, LifeAfter90 Study

**DOI:** 10.1002/alz.092148

**Published:** 2025-01-09

**Authors:** Yi Lor, Alexander Ivan B. Posis, Kristen M. George, Rachel Peterson, Paola Gilsanz, María M. M. Corrada, Rachel A. Whitmer

**Affiliations:** ^1^ University of California, Davis, Davis, CA USA; ^2^ University of Montana, Missoula, MT USA; ^3^ Kaiser Permanente Northern California Division of Research, Oakland, CA USA; ^4^ University of California, Irvine, Irvine, CA USA

## Abstract

**Background:**

Appropriate methods for age adjustment in associations of neuroimaging and late‐life cognition is not well defined, especially among those 90+ years old. We examined how different age adjustment approaches affect the associations of MRI/PET‐based regional brain markers and cognitive change in the oldest‐old.

**Method:**

*LifeAfter90* is a diverse cohort study of adults ages 90+ in Northern California. We evaluated the associations of volumetric MRI/PET brain regions of interest (ROI) with change in executive function and verbal episodic memory over 8 visits (∼6 months apart) using linear mixed models. The cognitive domains were assessed with the Spanish and English Neuropsychological Assessment Scales. All ROI were adjusted for total cranial volumes while free water, fractional anisotropy, and amyloid standardized uptake ratio (SUVR) were not. Age‐adjustment was assessed using five approaches: 1) no adjustment, 2) residual age at MRI scan estimated from regression models on total cranial volume, 3) within model adjustment for age at baseline cognitive interview, 4) age at MRI scan, and 5) age at both baseline interview and MRI scan. All models adjusted for practice effects and interview mode (telephone/in‐person).

**Result:**

Among 209 participants (mean age at baseline cognition = 91.7±2.0, mean age at imaging = 93.2±2.2), 58% were female, and 74% had some college or more. Older age at baseline cognitive assessment was associated with larger volumes in certain ROI (hippocampus, parietal, third ventricle), but lower in other ROIs (cerebrum gray, frontal) (Figure 1). Older age was also associated with higher white matter hyperintensities (WMH), free water, and amyloid SUVR. Adjustment for age using approaches 2‐5 resulted in no significant changes in the estimated association for each measure of ROI (e.g. hippocampus, third ventricle, WMH, amyloid SUVR) with baseline cognition and longitudinal cognitive change compared to approaches 1 (Figure 2). Model fit statistics (AIC, BIC, and log‐likelihood) indicate that approach 1 yielded the best fit.

**Conclusion:**

Age adjustment does not meaningfully change the magnitude of the estimated association of neuroimaging biomarkers with baseline cognition and cognitive change. In those 90+, adjustment for age in models may not be necessary potentially due in part to the narrow age range and smaller age variation in this group.